# Releation between marital adjustment and somatic symptoms on parents of children with Spina Bifida

**DOI:** 10.1192/j.eurpsy.2023.1229

**Published:** 2023-07-19

**Authors:** P. Ulual, V. Ozer, R. Çetiner, I. Alataş, O. Güçlü

**Affiliations:** 1 Istanbul Basaksehir Cam ve Sakura Sehir Hastanesi; 2 İstanbul Eğitim ve Araştırma Hastanesi; 3 İstanbul Bilim Üniversitesi, İstanbul, Türkiye

## Abstract

**Introduction:**

Spine Clearance; which means; SB is a congenital disease . In the first month of pregnancy, the unborn baby’s spine does not close properly is the result . In the spinal cord due to a developmental disorder often causes serious permanent disability . Multi-system associated with this congenital anomaly affected, families with infants and these infants are faced with major challenges in the future. Often the expectations of all parents is to have a normal, healthy children. The birth of a disabled child in the family, family members, their (lives, feelings , behaviors , social life) is a condition that affects negatively.

**Objectives:**

The SCL-90 is a self repost clinical rating scale oriented toward the symptomatic behavior of psychiatric outpatients. The primary symptom dimensions measured by the SCL-90 are the nine symptom constructs given below: somatization, obsessive-compulsive, interpersonal sensitivity, depression, anxiety, hostility, phobic anxiety, paranoid ideation, psychotism. Marital adjustment test: A 15-item scale that measures marital satisfaction. It was initially used to differentiate well-adjusted couples from distressed (unsatisfied) couples. The 15 items are answered on a variety of response scales.

**Methods:**

In this study; The SCL-90 and Marital Adjustment Test have been applied on parents of children with Spina Bifida. A total of 40 person was used which is 25 women and 15 men. Above of 1 score has been examined high level for SCL-90 test. For Marital Adjustment Test, we accept the lowest score is 1 and the highest score is 60. Cut-off score accepted is 43. If the parents have score that is below 43, is determined incompatible, and is above 43 is determined compatible.

**Results:**

The result has been obtained, showed us, the parents who have above 1 score for SCL-90, have below score 43 on Marital Adjustment Test.

**Conclusions:**

The study show that parents who have somatization problems are not align with their partners. Participants have symptomatic problems because of their children health situation can cause an unaligned marriage. This suggests that parents of patients with diseases like SB should get the needed psychiatric help and supportive care during the course of treatment.

**Disclosure of Interest:**

None Declared

